# Communication partner training for aged‐care workers: A scoping review

**DOI:** 10.1111/1460-6984.70016

**Published:** 2025-02-20

**Authors:** Bridget Burton, Kirstine Shrubsole, Asmita Manchha, Michelle King, Sarah J. Wallace

**Affiliations:** ^1^ The Queensland Aphasia Research Centre, School of Health and Rehabilitation Sciences The University of Queensland Herston Australia; ^2^ Surgical Treatment and Rehabilitation Service (STARS) Education and Research Alliance The University of Queensland and Metro North Hospital and Health Service Herston Australia

**Keywords:** aged care, ageing, communication, training

## Abstract

**Background:**

In aged‐care settings, direct care staff play a crucial role in supporting older people with communication needs. Many direct care staff, however, have unmet skill needs in interpersonal, intercultural, and intergenerational communication. Communication Partner Training (CPT) provides a potential solution. However, it is not known if existing programs address the diverse communication needs encountered in aged‐care settings. We sought to identify the key features of existing CPT programs to determine their suitability for the Australian aged‐care context.

**Aims:**

To identify existing CPT programs relevant to aged‐care settings and to describe their content and format.

**Methods:**

A scoping review was conducted in alignment with the Joanna Briggs Manual for Evidence Synthesis and Preferred Reporting Items for Systematic Reviews and Meta‐Analyses for Scoping Reviews reporting guidelines. Using a systematic search, we identified peer‐reviewed articles from five electronic databases: PubMed, PsycINFO, Embase, Cochrane and CINAHL. All retrieved articles were screened by title and abstract; 20% were independently screened by a second reviewer. All full‐text articles were independently assessed by two reviewers. Data describing the content and format of identified CPT programs wa**s** extracted using the Intervention Taxonomy and an author‐developed tool.

**Main Contribution:**

This review highlights critical gaps in existing CPT programs for aged‐care settings. Identified programs were predominantly disorder‐specific (79%), with the vast majority focusing on conditions like dementia or aphasia and failing to address broader communication needs arising from personal, social and environmental factors. Notably, no programs addressed intercultural communication, despite known cultural and linguistic diversity among aged‐care workers and recipients in countries such as Australia. Furthermore, few (9%) included intergenerational communication considerations. Most programs relied on in‐person delivery methods (67%), often led by health professionals (71%), which may be impractical for resource‐constrained and geographically dispersed aged‐care services. Furthermore, reported outcome measures varied (187 across 90 articles), and few evaluated both trainee and client (the ‘dyad’) outcomes. These findings underscore the need for comprehensive, scalable and contextually relevant CPT programs to address the complex communication challenges seen in aged‐care settings.

**Conclusions:**

There is a need for a comprehensive CPT program that is fit‐for‐purpose for direct care staff in aged‐care settings. This program should address the multifaceted and intersecting communication support needs of aged‐care recipients, including intercultural and intergenerational communication differences. The program should also incorporate resource‐feasible delivery methods and evaluate dyadic communication outcomes. Closing these gaps is vital to enhancing quality of care and life for older adults in aged‐care settings.

**WHAT THIS PAPER ADDS:**

**What is already known on the subject**

- Many older Australians have complex, unmet communication support needs. In aged‐care settings, direct care staff play a crucial role in supporting older people to communicate. Many aged‐care workers, however, have their own unmet skill needs in interpersonal, intercultural and intergenerational communication. Communication partner training (CPT) provides a potential solution. However, it is not known if existing programs address the diverse communication support needs encountered in aged‐care settings.

**What this paper adds to existing knowledge**

- Using a scoping review methodology, we identified and described CPT programs for direct support staff who work with older adults. Most CPT programs were disorder‐specific and targeted communication needs in dementia or aphasia. As aged‐care recipients have multifaceted, complex and intersecting communication support needs, these programs may fall short of addressing the training needs of aged‐care workers. The mode of training delivery was predominately in‐person and health professional‐led. Such delivery modes may not support implementation given resource constraints across the Australian aged‐care sector. CPT that can be feasibly implemented in aged‐care settings to address complex and intersecting communication support needs is required.

**What are the potential or actual clinical implications of this work?**

- Our analysis highlights the need for comprehensive, disorder‐agnostic CPT tailored for aged‐care staff to address diverse communication support needs. This CPT should consider scalable delivery modes effective across Australia's vast geography and limited trainer availability. It must include outcome measures reflecting the perspectives of communication support partners—older Australians in aged care with communication challenges. To tackle the health, personal and environmental communication barriers in aged care, a fit‐for‐purpose CPT program, co‐designed with key stakeholders, is essential to ensure the training is both feasible and acceptable to meet the unmet needs of intended end‐users.

## INTRODUCTION

Communication is a human right (United Nations General Assembly, 1948). It is fundamental to personhood and is intricately woven into almost all aspects of life (Owens et al., 2014). Through communication, we can make decisions, share our preferences, and meaningfully participate in daily activities of our choosing (Stipinovich et al., 2023). Despite this, communication is often underappreciated until challenges arise. In Australia, approximately 750 000 older adults live with a communication disability (Australian Institute of Health and Welfare, 2022a). Communication difficulties can impact an individual's ability to understand others and be understood (Speech Pathology Australia, 2024). They may arise from a range of pathological and non‐pathological causes (Worrall & Hickson, 2003; Yorkston et al., 2010), as well as from cultural and linguistic differences (Federation of Ethnic Communities' Councils of Australia, 2015), age‐related sensory changes (Guthrie et al., 2018), inter‐generational differences (Eull et al., 2023; Williams et al., 2023, 2003) and environmental barriers (Hickson et al., 2005). These factors often intersect (Manchha et al., 2024) and can collectively be described as ‘communication support needs’ (Speech Pathology Australia, 2024). Older adults with communication support needs are more likely to report a lower quality of life and experience negative psychological effects, such as depression, isolation and loneliness, compared to those without communication difficulties (Ciorba et al., 2012; Cruice et al., 2010, 2005; Hockley et al., 2023; Palmer et al., 2019; Spaccavento et al., 2014; Stransky et al., 2018; Yorkston et al., 2010). Due to challenges in comprehending and expressing personal preferences and choices, individuals with communication support needs are also at increased risk of being excluded from decision‐making (Bennett et al., 2020; Mattos et al., 2022). This is particularly the case for older adults who access aged‐care services (Speech Pathology Australia, 2023).

In Australia, approximately 1.3 million older adults currently access aged‐care services (Aged Care Quality and Safety Commission, 2024; Australian Institute of Health and Welfare, 2023b), including community‐based services, such as the Commonwealth Home Support Programme and Home Care Package schemes, as well as residential care services. Communication support needs are commonly reported among aged‐care recipients (Bennett et al., 2020; Jack et al., 2019; Older Persons Advocacy Network, 2023; Speech Pathology Australia, 2023; Yorkston et al., 2010). In residential aged‐care settings, where a person's support needs are often the highest, it is estimated that 95% of residents have at least one communication impairment (Bennett et al., 2016; Speech Pathology Australia, 2023; Worrall et al., 1993), and over half (54%) are living with dementia (Australian Institute of Health and Welfare, 2024a). Additionally, aged‐care recipients often face multiple, compounding comorbidities (Australian Bureau of Statistics, 2024; Guthrie et al., 2018), as well as personal and environmental factors, that further complicate their communication needs (such as age, cultural and linguistic diversity, barriers in the physical environment and societal attitudes) (Manchha et al., 2024). As a result, aged‐care recipients can encounter significant barriers to participating in conversations about their care, particularly if their communication needs are not adequately supported.

As communication is a two‐way process, it is essential that immediate communication partners, such as caregivers, are equipped to effectively support older adults' communication needs (Wiseman‐Hakes et al., 2020). For many aged‐care recipients, direct care staff are considered primary communication partners (Eriksson et al., 2016). In aged‐care settings, direct care staff include nursing staff (including both registered and enrolled nurses who provide medical and healthcare to aged‐care recipients), as well as personal care workers (who provide support with activities of daily living) (Aged Care Quality and Safety Commission, 2023; Australian Institute of Health and Welfare, 2022b). Communication is an essential activity for direct care staff (Martyn et al., 2022), with research indicating that personal care workers spend around 2 h per shift providing social and instrumental communication support to residents as part of their care role (Prgomet et al., 2017; Qian et al., 2014). Effective interactions are therefore dependent on communication partners being both knowledgeable and skillful in providing communication support (Conway & Chenery, 2016). In particular, personal care workers may have higher training needs, as they are often younger, have less formal educational qualifications, and many are from culturally and linguistically diverse backgrounds (Australian Department of Health and Aged Care, 2024)—factors that may impact communication. High attrition rates among personal care workers also impact the consistency of care provided, including knowledge and understanding of a care recipient's communication needs and preferences (McKenzie et al., 2024). Furthermore, in Australia, direct care staff may not receive specific or consistent training in communication support skills (Martyn et al., 2022; McKenzie et al., 2024). Given the unique needs of direct care staff, and evidence to suggest that even brief communication training interventions reduce caregiver burden and improve care‐based interactions (Sprangers et al., 2015), an evaluation of the suitability of existing programs for aged‐care settings is warranted.

Communication Partner Training (CPT) is an umbrella term describing a broad group of interventions that aim to improve interactions for people with communication support needs (Folder et al., 2024; Simmons‐Mackie et al., 2016; Wiseman‐Hakes et al., 2020). Most CPT programs are designed to be delivered to primary communication partners, such as family members, formal carers or frontline health professionals (Tessier et al., 2020). During CPT, communication partners are educated about communication needs and taught a range of strategies, techniques and environmental modifications that support communication interactions (Simmons‐Mackie et al., 2016; Tessier et al., 2020). There is evidence that CPT is efficacious in improving staff knowledge and confidence in providing patient‐centred care (Sprangers et al., 2015), as well as being a cost‐effective method of reducing incidents of verbal aggression and resistiveness to care (Williams et al., 2017). Implementing CPT for aged‐care workers promotes inclusive communication practices (Bennett et al., 2016; Speech Pathology Australia, 2024), and may also improve quality of care and healthcare outcomes for aged‐care recipients by promoting supported decision‐making (Bennett et al., 2020; Ervin et al., 2017; McCabe et al., 2021; McKenzie et al., 2024).

CPT programs are often developed to address support needs associated with specific conditions, such as dementia (Folder et al., 2024) and communication impairments caused by stroke (Simmons‐Mackie et al., 2016). Little research has explored the common and core elements of CPT programs across diagnostic groups (O'Rourke et al., 2018; Tessier et al., 2020). It is therefore not known if programs exist that address the complex and intersecting communication support needs experienced by aged‐care recipients, or the unique training needs of direct care staff.

Therefore, this review sought to explore the delivery methods, content and format of existing CPT programs for older adults with communication support needs. Our research question was: ‘What CPT programs exist for direct support staff who work with older adults?’ Our research aims were: (1) to identify existing CPT programs that have been designed for staff who work with older adults and (2) to describe the content and format of identified programs.

## METHODS

This scoping review was conducted in alignment with both the Joanna Briggs Manual for Evidence Synthesis (Aromataris et al., 2024) and the Preferred Reporting Items for Systematic Reviews and Meta‐Analyses Extension for Scoping Reviews (PRISMA‐ScR) (Tricco et al., 2018).

### Search strategy

Five databases were searched on 14 November 2023: PubMed, PsycINFO, Embase, Cochrane and CINAHL. A systematic search strategy was devised with assistance from a research librarian at the University of Queensland. The search strings used by O'Rourke et al. (2018) and Tessier et al. (2020) were expanded to include a broader range of communication disorders and differences relevant to older adults in aged‐care settings. For example, search terms such as, ‘hearing disorder’, ‘presbycusis’, ‘aged care’, ‘cultural and linguistic diversity’, and ‘elderspeak’ were included in the updated search strings (see Supplementary File S1). Records returned from database searches were collated and imported into Covidence (a reference management software). Duplicates were removed.

### Inclusion criteria

Records were included if: (1) they reported the evaluation, or implementation of an education or training program that focussed on communication skills development and/or communication partner training; (2) the reported training was delivered to healthcare professionals, aged‐care workers, students or formal carers; (3) the training related to older adult populations with communication support needs; and (4) the study was reported in English. Records reporting the development of training programs were excluded. Records reporting training programs delivered to family members and/or friends, or intended for paediatric populations, were also excluded. A detailed outline of the eligibility criteria can be seen in Supplementary File S1.

### Screening and data extraction

**FIGURE 1:**
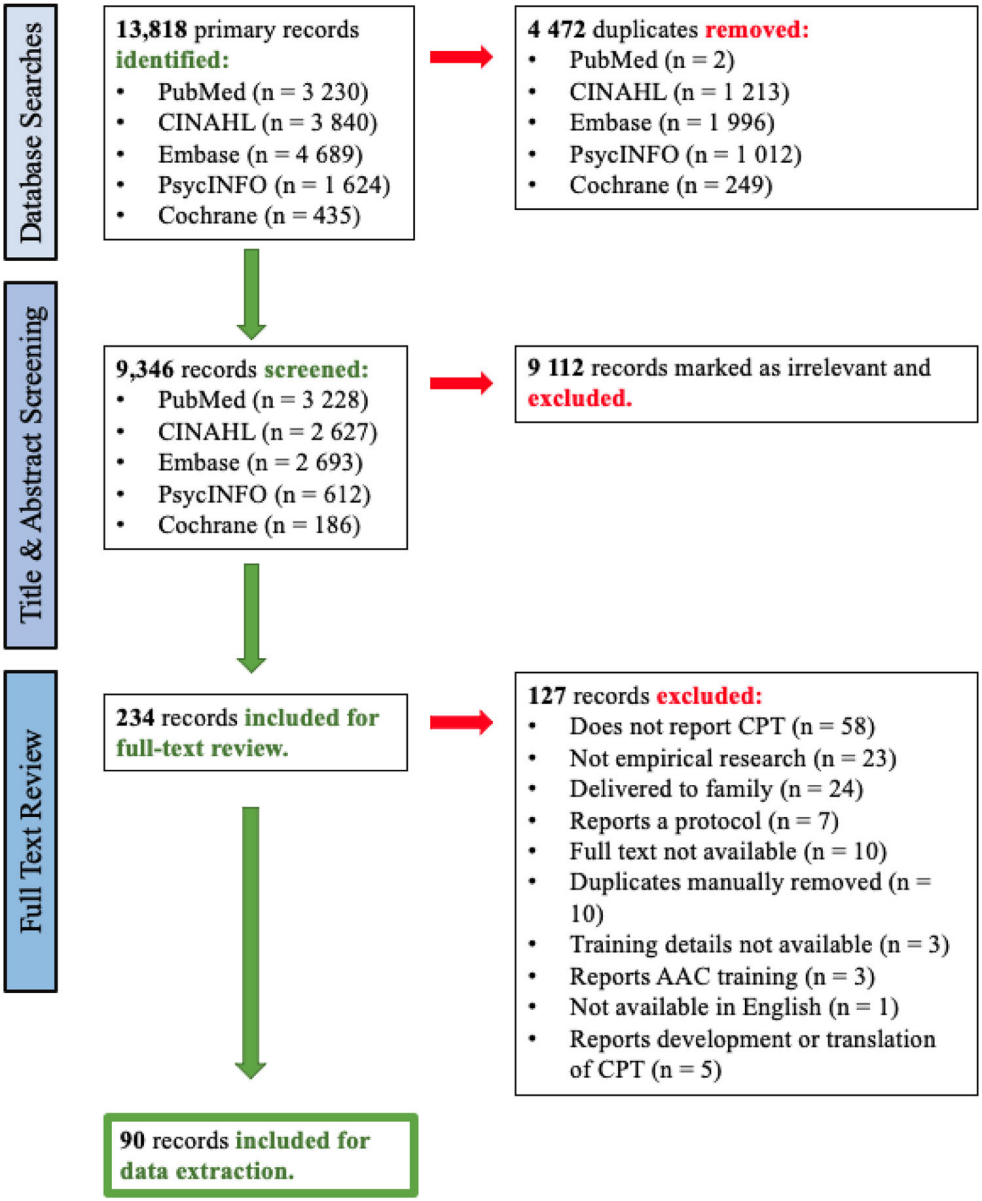
Preferred Reporting Items for Systematic Reviews and Meta‐Analyses for Scoping Reviews flow chart. Abbreviations: AAC, augmentative and alternative communication; CPT, Communication Partner Training.

Records were screened by the first and third authors. The first author (reviewer 1) is a speech pathologist with expertise and clinical experience in communication disability. The third author (reviewer 2) is an aged‐care researcher with expertise in workforce development and organisational psychology. Reviewer 1 completed title and abstract screening for all records using Covidence systematic review software. Reviewer 2 completed title and abstract screening for a random selection of 20% of records (*n* = 1870 records). Conflicts (*n* = 59) were resolved through discussion. In instances where the reviewers were unable to agree, the resolution was sought from a third reviewer (the second author). Cohen's Kappa was calculated to determine the level of agreement between reviewers 1 and 2 (*k* = 0.81; 0.80–0.90 indicating ‘strong agreement’) (McHugh, 2012). Following the completion of screening, reviewers 1 and 2 completed full‐text reviews of all remaining records (*n* = 234). The selection process is illustrated in Figure 1.

A Microsoft Excel template was used to manage data extraction. Data extraction was guided by two tools; (1) the Intervention Taxonomy (ITAX) (O'Rourke et al., 2018; Schulz et al., 2010) and (2) an author‐developed extraction tool. The ITAX provides a consistent framework for identifying, classifying and describing essential elements of intervention studies (Schulz et al., 2010). As per O'Rourke et al. (2018), the ITAX was selected as a data collection tool due to its comprehensive framework for describing intervention delivery characteristics. Specifically, the ITAX was used to collect information about intervention delivery mode and method, materials and activities, location, schedule and duration, participant and interventionist characteristics, program adaptability and implementation/fidelity from each article (Schulz et al., 2010). Information regarding CPT program scripting was not synthesised as most CPT program manuals could not be retrieved. In addition, an author‐developed data extraction tool was created to capture intervention characteristics that were not already accounted for by the ITAX. This included the citation and reference (including the year of publication), the name of the training program (if stated), the target population, research design, research aims, outcome measures and the overarching program contents.

### Data analysis and interpretation

Descriptive statistics and frequency counts were conducted for (1) year of publication, (2) research design, (3) communication support needs addressed, (4) training programs, (5) mode of delivery, (6) materials and activities, (7) delivery setting, (8) schedule and length of intervention, (9) participant and interventionist characteristics and (10) adaptability and fidelity. Reviewer 2 (the third author) then cross‐checked the extracted data points listed in Table 1 for all 90 articles. Finally, outcomes measured across all articles were collated and subsequently categorised as (1) training recipient outcomes, (2) care recipient outcomes, (3) service outcomes or (4) other (refer to Supplementary File S1). Outcomes were then classified into construct domains. For example, all outcomes that measured change in a trainee's understanding of communication difficulties and/or ways to support communication needs were classified under the domain ‘Knowledge’.

**TABLE 1:** Article characteristics.

Article number and citation	Name of CPT program reported	Research design	CPT Area of Focus	Delivery setting	Interventionist	Trainee
1. Conway and Cherney (2016)^a^	MESSAGE Communication Strategies for Dementia	RCT	Dementia	Community aged care	Speech pathologist	Personal care workers
2. Broughton et al. (2011)^a^	MESSAGE Communication Strategies for Dementia (& RECAPS)	Pre/post (control)	Dementia	Residential aged care	Speech pathologist and psychologist	Nursing staff
3. Irvine et al. (2003)^a^	Interactive Multimedia Program (‘Speaking Skills, Reacting Skills, Redirection and Communication Cards’)	Pre/post (control)	Dementia	Residential aged care	Researcher	Personal care workers
4. O'Brien et al. (2018)	VideOing to Improve Communication through Education (VOICE)	Pre/post (no control)	Dementia	Hospital (unspecified)	Speech pathologist	Health professionals
5. Pilnick et al. (2023)	Conversation Analysis Based Simulation (CABS) (an extension of the VOICE program)	Pre/post (no control)	Dementia	Hospital (acute)	Speech pathologist	Health professionals
6. McCallion et al. (1999) ^a^	Nursing Assistant Communication Skills Program (NACSP)	Pre/post (control)	Dementia	Residential aged care	Social worker	Nursing staff
7. Weitzel et al. (2011)	NICHE Educational Intervention	Pre/post (no control)	Dementia	Hospital (inpatient)	*Not specified*	Nursing staff
8. Sanchez‐Martinez et al. (2023)^a^	The Validation Method	Mixed methods	Dementia	Residential aged care	*Not specified*	Health professionals
9. Soderlund et al. (2016)^a^	The Validation Method	Qualitative	Dementia	Residential aged care	*Not specified*	Nursing staff
10. Ripich (1994)^a^	FOCUSED Training Program	Pre/post (no control)	Dementia	Residential aged care	*Not specified*	Nursing staff
11. Ripich et al. (1995)^a^	FOCUSED Training Program	Pre/post (no control)	Dementia	Residential aged care	Speech pathologist and nurse	Nursing staff
12. Savundranayagam et al. (2020)^a^	Be ‘EPIC’ [The Environment, Using Person Centred Communication, Focusing on Relationships (‘I matter too’) and Incorporating Clients Abilities].	Qualitative	Dementia	Residential aged care	*Not specified*	Personal care workers
13. Savundranayagam et al. (2021)^a^	Be ‘EPIC’ [The Environment, Using Person Centred Communication, Focusing on Relationships (‘I matter too’), and Incorporating Clients Abilities].	Qualitative	Dementia	Residential aged care	*Not specified*	Personal care workers
14. Chao et al. (2016)^a^	Advanced Innovative Internet‐based Communication Education (AIICE)	Repeated measures	Dementia	Residential aged care	*Not specified*	Nursing staff
15. Passalacqua & Harwood (2012)^a^	VIPS Communication Skills Training	Pre/post (no control)	Dementia	Residential aged care	Researcher	Personal care workers
16. Smyth et al. (2023)^a^	Adapted from: The VERA Communication Skills Framework Training	Qualitative	Dementia	Residential aged care	Researcher	Students
17. Naughton et al. (2018a)	The VERA Communication Skills Framework Training	Other	Dementia	Hospital (acute)	Researcher	Students
18. Naughton et al. (2018b)	The VERA Communication Skills Framework Training	Pre/post (control)	Dementia	Hospital (acute)	Researcher	Students
19. Sprangers et al. (2015)^a^	Hybrid of The Communication Enhancement Model, FOCUSED, and Come Talk with Me	Pre/post (control)	Dementia	Residential aged care	*Not specified*	Nursing staff
20. Beer et al. (2012)	Communicating with People Who Have Advanced Dementia	RCT	Dementia	Community setting	Music therapist	Students
21. Levy‐Storms et al. (2016)^a^	*Name of training not stated*.	Pre/post (no control)	Dementia	Residential aged care	*Not specified*	Nursing staff
22. Magai et al. (2002)^a^	Nonverbal Sensitivity Training Program	RCT	Dementia	Residential aged care	Psychologist	Personal care workers
23. Burgio et al. (2001)^a^	Come Talk With Me	Pre/post (control)	Dementia	Residential aged care	Psychologist	Nursing staff
24. Bourgeois et al. (2004)^a^	Come Talk With Me	Pre/post (control)	Dementia	Residential aged care	Psychologist	Nursing staff
25. Williams and Gurr (2016)	Adapted from: On‐the‐Spot Training	Pre/post (no control)	Dementia	Hospital (acute)	Psychologist	Nursing Staff
26. Franzmann et al. (2016)^a^	TANDEM (and MultiTANDEM)	Pre/post (control)	Dementia	Residential aged care	Nurses and social workers	Nursing staff
27. Degen et al. (2022)^a^	TANDEM Communication Training for Caregivers of People with Dementia	Pre/post (control)	Dementia	Residential aged care	*Not specified*	Nursing staff
28. Douglas et al. (2023)^a^	Dementia Collaborative Coaching (‘a Communication Coaching Intervention’)	Pre/post (no control)	Dementia	Residential aged care	Speech pathologist and nurse	Nursing staff
29. Kagan et al. (2001)	SCA	RCT	Acquired Communication Disorder	Community setting	Speech pathologist	Volunteers
30. van Rijssen et al. (2019)	Adapted from SCA	Pre/post (no control)	Aphasia	Hospital (inpatient)	Speech pathologist and& researcher	Nursing staff
31. Kagan (1998)	SCA	Other	Aphasia	Community setting	Speech pathologist	Health professionals
32. Rayner and Marshall (2003)	SCA	Other	Aphasia	Community setting	Speech pathologist	Volunteers
33. Hansen et al. (2020)	Adapted from SCA	Qualitative	Aphasia	Hospital (inpatient)	Speech pathologist	Health professionals
34. Kagan et al. (2018)	SCA	RCT	Aphasia	Community setting	Speech pathologist	Volunteers
35. Jensen et al. (2014)	Adapted from SCA	Mixed methods	Aphasia	Hospital (acute)	Speech pathologist	Nursing staff
36. Power et al. (2023)	Adapted from SCA	Pre/post (no control)	Aphasia	University setting	Speech pathologist	Students
37. Power et al. (2020)	Adapted from SCA	RCT	Aphasia	University setting	Speech pathologist	Students
38. Heard et al. (2017)	Adapted from SCA	RCT	Aphasia	Hospital (inpatient)	Speech pathologist	Health professionals
39. Wilkinson et al. (2013)	Adapted from SCA	Pre/post (no control)	Aphasia	University setting	Speech pathologist	Students
40. Coelho de Matos et al. (2023)	Adapted from SCA	Pre/post (no control)	Stroke	University setting	Speech pathologist and physiotherapist	Health professionals
41. Nielsen et al. (2023)	Adapted from SCA	Pre/post (no control)	Traumatic brain injury	Hospital (inpatient)	Speech pathologist	Health professionals
42. Simmons‐Mackie et al. (2007)	Adapted from SCA	Qualitative	Aphasia	Hospital (multiple wards)	Speech pathologist	Health professionals
43. Welsh and Szabo (2011)	Adapted from SCA	Pre/post (no control)	Aphasia	Community setting	Speech pathologist	Students
44. van Rijssen et al. (2021)	CommuniCare	Qualitative	Aphasia	Hospital (inpatient)	Speech pathologist and researcher	Health professionals
45. Brock and Scharp (2020)	Connect: The Communication Disability Network—Making Communication Access a Reality	RCT	Aphasia	University setting	Speech pathologist	Students
46. Lee et al. (2020)	Connect: The Communication Disability Network—Making Communication Access a Reality	Pre/post (no control)	Aphasia	University setting	Speech pathologist	Students
47. Finch et al. (2017)	Connect: The Communication Disability Network—Making Communication Access a Reality	RCT	Aphasia	University setting	Speech pathologist	Students
48. Finch et al. (2020)	Connect: The Communication Disability Network—Making Communication Access a Reality	Mixed methods	Aphasia	University setting	Speech pathologist	Students
49. Finch et al. (2018)	Connect: The Communication Disability Network—Making Communication Access a Reality	RCT	Aphasia	University setting	Speech pathologist	Students
50. Shrubsole et al. (2021)	Connect: The Communication Disability Network—Making Communication Access a Reality	Implementation study	Aphasia	Hospital (inpatient)	Speech pathologist	Health professionals
51. Horton et al. (2016)	Connect: The Communication Disability Network—Making Communication Access a Reality	Qualitative	Aphasia	Hospital (inpatient)	Speech pathologist	Health professionals
52. Cameron et al. (2017)	Connect: The Communication Disability Network—Making Communication Access a Reality	Pre/post (no control)	Aphasia	Hospital (unspecified)	Speech pathologist	Health professionals
53. Cameron et al. (2018)	Connect: The Communication Disability Network—Making Communication Access a Reality	Qualitative	Aphasia	University setting	Speech pathologist	Students
54. Cameron et al. (2019)	Hybrid of: Connect: The Communication Disability Network X Supported Conversation for Adults with Aphasia	RCT	Aphasia	Hospital (unspecified)	Speech pathologist	Health professionals
55. Nikkels et al. (2023)	Training ContAct (A Dutch Communication Partner Training)	Mixed methods	Aphasia	University setting	Speech pathologist	Students
56. Hickey et al. (2004)^a^	*Name of training not stated*.	Case study	Aphasia	Residential aged care	Speech pathologist	Students
57. Horton et al. (2016)	Supporting Communication for Access and Participation (‘SC Skills Training’)	Pre/post (control)	Aphasia	Hospital (inpatient)	*Not specified*	Health professionals
58. Lin et al. (2022)	*Name of training not stated*.	Pre/post (no control)	Aphasia	University setting	Speech pathologist	Students
59. Gaeta and Sharpp (2023)	IPE (Interprofessional Education) for Communication Sciences and Disorders Program	Pre/post (no control)	Hearing Impairment	University setting	Speech pathologist and nurse	Students
60. Palmer et al. (2017)^a^	HearCARE Training (Hearing and Communication Assistance for Resident Engagement)	Case study	Hearing Impairment	Residential aged care	Audiologist	*Not specified*
61. McShea and Ferguson (2023)^a^	C2Hear Training (modified to be ‘Hearing Champion Training’)	Mixed methods	Hearing Impairment	Residential aged care	Audiologist	Personal care workers
62. Sanchez et al. (2017)	*Name of training not stated*.	Pre/post (no control)	Hearing Impairment	Community setting	*Not specified*	Health professionals
63. Williams et al. (2017)^a^	CHAT	RCT	Elderspeak	Residential aged care	Researcher	Nursing staff
64. Williams (2006)^a^	CHAT	Repeated measures	Elderspeak	Residential aged care	Researcher	Nursing staff
65. Coleman et al. (2015)^a^	CHAT	Pre/post (control)	Elderspeak	Residential aged care	*Not specified*	Nursing staff
66. Williams et al. (2016)^a^	CHAT	Pre/post (control)	Elderspeak	Residential aged care	*Not specified*	Nursing staff
67. Williams et al. (2023)^a^	CHATO (based on CHAT)	Mixed methods	Elderspeak	Community aged care	*Not specified*	Nursing staff
68. Williams et al. (2021)^a^	CHATO (based on CHAT)	RCT	Elderspeak	Residential aged care	*Not specified*	Nursing staff
69. Coleman et al. (2022)^a^	CHATO (based on CHAT)	RCT	Elderspeak	Residential aged care	*Not specified*	Nursing staff
70. Tessier et al. (2021)	Accessible Communication in Public Transportation	Pre/post (no control)	Communication disorders and disabilities	Community setting	Speech pathologist	Public servants
71. Saldert et al. (2016)	FRAME Program	Pre/post (control)	Communication disorders and disabilities	University setting	Speech pathologist	Students
72. Baylor et al. (2019)	FRAME Program	Pre/post (no control)	Communication disorders and disabilities	University setting	Speech pathologist	Students
73. Mach et al. (2022)	FRAME Program	Pre/post (no control)	Communication disorders and disabilities	University setting	Speech pathologist	Students
74. Forsgren et al. (2017)	FRAME Program	Pre/post (no control)	Acquired Communication Disorder	University setting	Speech pathologist	Students
75. Bryan et al. (2002)^a^	Speakability Training (previously named ‘Communicate’ Training)	Pre/post (control)	‘Older adult’ communication	Community aged care	Speech pathologist	Personal care workers
76. Maxim et al. (2001)^a^	Communicate Training	Other	Communication disorders and disabilities	Both residential and community aged care	Speech pathologist	Nursing staff
77. Eriksson et al. (2016)^a^	Adapted from: Supporting Partners of People with Aphasia in Relationships and Conversation (SPPARC)	Pre/post (no control)	Communication disorders and disabilities	Residential aged care	Speech pathologist	Nursing staff
78. Forsgren and Saldert (2022)^a^	Adapted from: Patient‐Centred Communication Intervention	Qualitative	Communication disorders and disabilities	Residential aged care	Speech pathologist	Nursing staff
79. Chu et al. (2018)	Interprofessional (IP) Communication Training Program (based on Patient‐Centred Communication Intervention)	Pre/post (no control)	Stroke	Hospital (inpatient)	Speech pathologist and nurse	Nursing staff
80. Sorin‐Peters et al. (2010)^a^	Patient‐Centred Communication Intervention	Pre/post (no control)	Communication disorders and disabilities	Long term care	Speech pathologist	Nursing staff
81. McGilton et al. (2011)^a^	Patient‐Centred Communication Intervention	Pre/post (no control)	Communication disorders and disabilities	Long term care	Speech pathologist	Nursing staff
82. Tate et al. (2020)	Student SPEACS—Study of Patient‐Nurse Effectiveness with Assistive Communication Strategies	Mixed methods	Communication disorders and disabilities	University setting	*Not specified*	Students
83. Al‐Wathinani et al. (2022)	*Name of training not stated*.	Pre/post (no control)	Communication disorders and disabilities	University setting	Speech pathologist	Students
84. Behn et al. (2012)	Adapted from: TBI Express	RCT	Traumatic brain injury	Hospital (inpatient)	Speech pathologist	Personal care workers
85. Behn et al. (2015)	Adapted from: TBI Express	Qualitative	Traumatic brain injury	Hospital (inpatient)	Speech pathologist	Personal care workers
86. Togher et al. (2004)	TBI Police Training Program	RCT	Traumatic brain injury	Community setting	Speech pathologist and researcher	Public servants
87. McKinley et al. (2015)	Supported Conversation Volunteer Program (Hybrid of Connect X SCA)	Qualitative	Acquired Communication Disorder	Hospital (acute)	Speech pathologist	Volunteers
88. Koski et al. (2010)^a^	OIVA (Finnish Communication Training Program)	Qualitative	Learning dsability	Long‐term care	Speech pathologist	Personal care workers
89. Thomas et al. (2014)	*Name of training not stated*.	Pre/post (no control)	Intellectual disability	University setting	Speech pathologist & psychiatrist	Students
90. Harper and Wadsworth (1992)	Making Contact: A Strategy to Train Healthcare Professional to Communicate with Adults with Mental Retardation	Pre/post (no control)	Intellectual disability	Research centre	*Not specified*	Volunteers

^a^ Articles reporting on CPT delivered in aged‐care settings.

Abbreviations: CHAT, Changing Talk Communication Training; CHATO, Changing Talk Online Communication Training; CPT, Communication Partner Training; RCT, randomised controlled trial; SCA, Supported Conversation for Adults with Aphasia; TBI, traumatic brain injury.

## RESULTS

Ninety journal articles were included in this scoping review (see Table 1 and Supplementary File S1). Articles were published between 1992 and 2023, with over half published between 2016 and 2023 (*n* = 57, 63%). The most frequently reported research design was pretest‐posttest, accounting for 50% (*n* = 45: 35% pretest‐posttest with no control; 16% pretest‐posttest with control). Other common research designs included randomised controlled trials (*n* = 16, 18%), qualitative research studies (*n* = 13, 14%) and mixed methods studies (*n* = 7, 8%).

### Communication support needs targeted by training programs

The articles were reviewed to identify the types of communication support needs targeted. Overall, 79% (*n* = 71) reported on disorder‐specific CPT programs, with dementia (*n* = 28, 31%) and aphasia (*n* = 27, 30%) most common. Other disorder‐specific programs targeted hearing impairment (*n* = 4, 5%), traumatic brain injury (*n* = 4, 5%), acquired communication disorder (*n* = 3, 3%), stroke (*n* = 2, 2%), intellectual disability (*n* = 2, 2%) and learning disability (*n* = 1, 1%). Eight articles (9%) focused on intergenerational communication: seven on ‘elderspeak’ CPT programs (8%) and one on ‘older adult’ communication skills (1%). No included articles reported on CPT relating to visual impairment or culturally and linguistically diverse populations.

### Distinct communication partner training programs identified

Forty‐five different CPT programs were identified across the 90 articles. The most common CPT program was Supported Conversation for Adults with Aphasia (SCA), (*n* = 15 articles, 17%). Other frequently studied CPT programs were Connect: The Communication Disability Network—Making Communication Access a Reality (*n* = 9 articles, 10%) and Changing Talk Communication Training (CHAT)/Changing Take Online (CHATO) (*n* = 7 articles, 8%). The 45 CPT programs can be viewed in Table 1.

## THE INTERVENTION TAXONOMY (ITAX)

### Dimension 1: Mode of intervention

In most articles, CPT programs were delivered in‐person, in a group‐based setting (*n* = 60, 67%) (see Supplementary File S1). Hybrid delivery of CPT programs (where training was delivered both in‐person and online) was reported in six (7%) articles. Other delivery modalities included in‐person, individual training (*n* = 4, 4%), lecture‐based learning (*n* = 4, 4%), asynchronous training through online modules and activities (*n* = 5, 6%) and online training via videoconference (*n* = 4, 4%).

### Dimension 2: Intervention materials

Dimension 2 presents data based on the 45 CPT programs. Here, we describe the training activities and materials required by programs. Results are summarised below with full details provided in the supplementary material.

**Materials**. The 45 CPT programs identified in the 90 articles used combinations of intervention materials (see Supplementary File S1). Twenty‐nine (60%) used DVDs or video vignettes to demonstrate communication strategies or patient‐carer interactions. Twenty‐three (48%) programs used in‐person presentation materials, and 16 (33%) reported having an interventionist training manual. Fifteen programs (31%) used participant activity sheets, workbooks and case scenarios, and 11 (23%) used paper‐based handouts. No materials were identified for four (9%) programs.

**Activities**. The 45 CPT programs had a range of activities, including theoretical, demonstration and practice activities. It was not always possible to extract the full content of a program from the text of the articles, so here we report on those program activities that could be identified for review. Almost all (*n* = 42/45, 88%) CPT programs specified that they contained a theoretical component. Theory could entail a combination of background to the communication disorder/population, specific communication challenges experienced by the individual and strategies to support or enhance interactions. Thirty‐one CPT programs (65%) included a video presentation to demonstrate patient–provider interactions or the use of communication support strategies. Over half (*n* = 27, 56%) of the CPT programs included direct feedback on the communication skills of trainees. Other common learning and practical activities included: group discussion and debriefing (*n* = 20, 42%), role‐play (*n* = 18, 38%) and direct involvement of a person living with communication support needs (*n* = 13, 27%).

### Dimension 3: Location of intervention

Of the 90 articles in this review, 38 (42%) reported CPT delivered in aged‐care settings. Twenty‐two articles (25%) delivered training in hospital settings. Other locations included educational settings such as universities (*n* = 21, 23%), and community settings (such as a police training academy, *n* = 9, 10%). Findings are displayed in Supplementary File S1.

### Dimension 4: Schedule of intervention

**Total Time Duration**. The reported duration of training varied considerably across the 90 articles: ranging between 12 min (Weitzel et al., 2011) and 39.25 h (van Rijssen et al., 2021). Thirty‐four articles (38%) specified that training was between 1 and 4 h in duration, and 33 (37%) described training longer than 4 h. Seventeen articles (19%) did not specify the duration of training.

**Delivery Schedule**. The most common delivery schedule for CPT was a single occasion of training (*n* = 31, 35%). The longest training program spanned over 2 years and 2 months (Koski et al., 2010), as it included multiple ‘booster’ and follow‐up sessions. Eighteen articles did not specify the schedule of delivery.

### Dimension 5: Scripting

We were unable to source the scripting of CPT training for all 45 programs delivered in the 90 included articles. Therefore, we are unable to describe details of CPT program scripting or analyse these further.

### Dimension 6: Participant characteristics

In the included articles, 30 (33%) delivered training to nursing staff, 25 (28%) to students (university and vocational health studies), 16 (18%) to health professionals (allied health and medical professionals) and 11 (12%) to care staff (paid carers and caregivers, support workers and personal care workers) (see Supplementary File S1). Training was also delivered to cohorts that were not from health or caring professions, including volunteers (*n* = 5, 6%) and public servants or council workers (*n* = 2, 2%). One article did not specify the professional background of the training recipients (Palmer et al., 2017).

### Dimension 7: Interventionist characteristics

The training was delivered by a health professional or a researcher in the majority of included articles (*n* = 71, 78%) (see Supplementary File S1). Nineteen (21%) articles did not specify who delivered the training. Speech pathologists were the most common health profession to deliver CPT (*n* = 45, 50%, one or more speech pathologists), with an additional seven articles (8%) focussing on training delivered by a speech pathologist alongside another health professional (such as a nurse or psychologist). Researchers were the next most common interventionists (*n* = 7, 8%), followed by psychologists (*n* = 4, 5%), then audiologists (*n* = 2, 2%).

### Dimension 8: Adaptability of program

Fifteen of the 90 articles (17%) outlined adaptations that were made to the CPT program delivered. Common adaptations included reducing information or modifying training content due to time restrictions (*n* = 7, 8%), varying the number of individual or group‐based follow‐up sessions depending on the educational needs of the trainees (*n* = 4, 4%), and modifying materials used based on the delivery setting (*n* = 2, 2%). The remaining articles (*n* = 75, 83%) did not report whether adaptations were made.

### Dimension 9: Implementation of intervention

**Fidelity**. Statements regarding implementation fidelity were identified in 13 articles. Methods of recording implementation fidelity included checklists or treatment adherence logs (*n* = 5, 6%), and the use of researcher field notes or data collection sheets (*n* = 4, 4%). In one article, authors reported only allowing trainees to progress if online modules were fully completed (Williams et al., 2023, 2021). In another, treatment fidelity was described as variable due to the inherent nature of using simulated actors for training (Pilnick et al., 2023). Power et al. (2020) described seeking to strengthen treatment fidelity by video‐recording a theoretical lecture component and using this for both online and in‐person training to ensure consistency.

### ITAX: Training programs specific to aged‐care settings

Less than half of the articles included in our review were specific to CPT delivered in aged‐care settings. Of these 38 articles, 34 delivered training in residential and/or long‐term aged‐ care (*n* = 34), and two in both community and residential aged care (*n* = 2). Two articles involved delivering training in community‐based aged care only (*n* = 2). In these 38 articles, 23 different CPT programs were identified. In this section, we report percentages based on the 38 aged‐care specific articles.

Of the 38 aged‐care specific articles, 21 articles (55%) described CPT that was designed to support people with dementia. These 21 articles described 15 different dementia‐specific CPT programs. Other specific communication support needs included people with general communication disabilities and disorders (*n* = 5, 13%), hearing impairments (*n* = 2, 5%), learning disabilities (*n* = 1, 3%) and aphasia (*n* = 1, 3%). Intergenerational communication (‘elderspeak’) CPT was also described in eight articles (21%).

Training programs were mostly delivered by speech pathologists (*n* = 12, 32%) or researchers (*n* = 5, 13%), although 14 articles did not specify the interventionist. The training was most often delivered to nursing staff or personal care workers (*n* = 34, 89%), with less commonly reported training recipients including students (*n* = 2, 5%) and health professionals (*n* = 1, 3%). Most training was delivered to trainees in‐person (*n* = 27, 71%), usually in a group‐based setting (*n* = 24, 63%), with two articles describing training individual trainees in‐person (Douglas et al., 2023; Palmer et al., 2017), and one article using a hybrid modality (both online and in‐person components) (Chao et al., 2016). Four articles described training delivered to aged‐care workers via an online asynchronous modality (Coleman et al., 2022; Irvine et al., 2003; Williams et al., 2023, 2021), and one article used ‘live’ online videoconferencing (Coleman et al., 2015).

## TRAINING OUTCOMES

Training outcomes were measured in a variety of ways across the 90 articles included in this review. We counted a total of 235 instances where outcomes had been measured. This included 187 different outcome measurement tools (see Supplementary File S1). Of the 235 outcome measures identified, 148 (63%) were classified as ‘training‐recipient outcomes’, 39 (17%) as ‘person‐specific outcomes’, four (2%) as ‘service outcomes’ and 44 (18%) as ‘other’. A full list of outcome measures and their aims, administration schedules and significant findings reported can be accessed in Supplementary File S1.

### Training recipient outcomes

**Knowledge**. Forty‐six outcome measures evaluated changes in trainee knowledge. This included changes to the trainee's reported knowledge of communication disorders or difficulties, as well as changes in knowledge of how to support an individual's specific communication needs. Changes in knowledge were typically measured through tests, questionnaires, or post‐training self‐report surveys. In one study (Simmons‐Mackie et al., 2007), changes in knowledge were assessed through qualitative measures, including post‐training individual interviews and focus group discussions.

**Skills**. Forty‐four outcome measures related to changes in the trainee's communication skills, behaviours and/or observed communicative interactions with care recipients. Communication checklists and rating scales were a common method for evaluating observed change. For example, the Communication Skills Checklist was used in four articles (Bourgeois et al., 2004; Burgio et al., 2001; Sprangers et al., 2015; van Rijssen et al., 2021) to evaluate the effectiveness of communicative interactions between patients and recipients, as well as their demonstrated use of communication support strategies and techniques. Other evaluation methods included ratings of pre‐post video recordings of patient‐recipient interactions, via the use of simulated patients (‘actors’), or role‐playing scenarios with real individuals with communication support needs while receiving real‐time feedback.

**Psychosocial**. Twenty outcome measures evaluated changes in trainee feelings or work satisfaction levels following training. Examples of measures relating to work satisfaction included the Burnout Inventory (Emotional Exhaustion and Depersonalisation Subscales) (Passalacqua & Harwood, 2012), The Modified Burden Scale (Behn et al., 2012), Perceived Ease of Caregiving (McGilton et al., 2011), the Interpersonal Reactivity Index (Empathetic Concern & Perspective Taking Subscales) (Passalacqua & Harwood, 2012) and the Occupational Mental Stress Scale (Franzmann et al., 2016).

**Confidence**. Thirteen outcome measures evaluated change in the trainee's perceived confidence (i.e., confidence in providing care to persons with communication support needs) post‐training. Five articles used questionnaires or surveys to measure the change in the trainee's confidence (Conway & Chenery, 2016; Irvine et al., 2003; McShea & Ferguson, 2023; O'Brien et al., 2018; Williams & Gurr, 2016). Similarly, seven articles used self‐reported rating scales, where trainees could indicate their own perceived levels of confidence after training (Baylor et al., 2019; Cameron et al., 2017; Finch et al., 2018; Heard et al., 2017; Mach et al., 2022; O'Brien et al., 2018; Williams et al., 2023).

**Attitudes**. Eleven outcome measures evaluated changes in trainee attitudes. Outcome measures typically related to changes in attitudes towards the specific population which was the focus of the training, such as towards people with dementia (Chao et al., 2016; Conway & Chenery, 2016; Passalacqua & Harwood, 2012). Three outcome measures evaluated attitudes to providing care to patients in general (Conway & Chenery, 2016; Tate et al., 2020). Another outcome measure related to changes in attitudes and perspectives of interprofessional collaboration following training (Gaeta & Sharpp, 2023).

**Multiple Domains**. Fourteen outcome measures evaluated multiple domains which included combinations of knowledge, attitudes and confidence. These measures were typically author‐developed and tailored to the training program (Beer et al., 2012; Cameron et al., 2019; Finch et al., 2020; Forsgren et al., 2017; Lin et al., 2022; Nielsen et al., 2023; Saldert et al., 2016).

### Care recipient outcomes

**Emotions and Behaviour**. Sixteen outcome measures related to changes in patient/resident emotions or behaviours. Several of these measures are related to either observed or reported levels of depression (Chao et al., 2016; Degen et al., 2022; Magai et al., 2002; McCallion et al., 1999; McGilton et al., 2011). One measure evaluated change in instances of psychotropic medication restraint use pre‐ and post‐training (McCallion et al., 1999).

**Communication Skills**. Ten outcome measures related to the change in the patient or resident's communication skills or communicative interactions. The most common outcome measure within this domain was the Measure of Participation in Conversation for Adults with Aphasia, which was included in five articles (Behn et al., 2012; Brock & Scharp, 2020; Kagan et al., 2001; Rayner & Marshall, 2003).

**Psychosocial Functioning**. Six outcome measures evaluated changes in psychosocial functioning of the patients/residents. This included quality of life assessments (e.g., the Stroke and Aphasia Quality of Life Scale‐39 g) (Horton et al., 2016; McGilton et al., 2011), evaluations of participation in activities of daily living (Degen et al., 2022; Horton et al., 2016), independent functioning (Burgio et al., 2001) and self‐esteem (Lee et al., 2020).

**Quality of Care**. Two measures gathered patient or resident perceptions of the quality of care provided by care staff (‘trainees’): the Relational Care Scale (McGilton et al., 2011), and the Communication Access Measure for Stroke (Horton et al., 2016).

**Effectiveness**. Patient/resident perceptions of training effectiveness were gauged using two measures, a verbal questionnaire (Lee et al., 2020) and an aphasia‐friendly survey (McKinley et al., 2015).

### Service outcomes

Four outcome measures evaluated change at the service level. Horton et al. (2016) included a service evaluation (a cost‐analysis) based on the EuroQol 5 Dimensions Health Questionnaire (three levels, the EQ‐5D‐3L), a health‐related quality‐of‐life outcome measure used for economic modelling and analysis. Staff attrition and turnover rates were evaluated in one article (McCallion et al., 1999), and a global evaluation of staff–patient relationships were included in another (McGilton et al., 2011). One study used a checklist to observe, document and analyse the physical communication environment of a facility (Simmons‐Mackie et al., 2007).

## DISCUSSION

This scoping review provides an overview of the common delivery modalities, elements and outcome measures used in CPT programs that are relevant to direct care staff who work with older adults with communication support needs. Our principal findings show that CPT programs were usually disorder or condition‐specific, with most relating to dementia or aphasia. Few articles considered communication issues more broadly for people using aged care, such as personal factors or social determinants. No articles were identified that described CPT for culturally and linguistically diverse populations. This potentially limits the utility of these programs in aged‐care settings where support needs are likely to be varied, concurrent, and changeable (Guthrie et al., 2018; Hickson et al., 2005; Yorkston et al., 2010). Further, CPT training in included studies was resource intensive, relying on in‐person delivery, usually by clinicians or subject matter experts. This model of training delivery may not be scalable across aged‐care services, particularly in regional, rural and remote geographical locations. Finally, the outcomes measured in the included studies were usually trainee‐specific, and the benefits of such programs for care recipients are therefore difficult to gauge. These key findings are explored next.

### Content of CPT programs

Our review did not identify any comprehensive CPT programs that considered intersecting communication support needs. Most articles (79%) included in this review described disorder‐specific training (such as for people living with dementia). This finding is consistent with Tessier et al. (2020) review, where 79% of their included articles reported a disorder‐specific program, predominantly for dementia, traumatic brain injury and communication impairments caused by stroke. Aged‐care recipients are likely to have greater levels of functional communication difficulties if they have multiple comorbidities, such as combinations of vision, hearing or cognitive impairments (Guthrie et al., 2018). In addition, aged‐care recipients have multilayered and complex communication support needs due to combinations of health, personal and environmental factors (Manchha et al., 2024; Worrall & Hickson, 2003). For these reasons, disorder‐specific CPT programs may fall short of meeting the training needs of direct care staff in aged‐care settings.

Our review also revealed that few CPT programs address personal factors or social determinants, such as intergenerational and intercultural considerations. For example, we did not identify any CPT programs relating to intercultural communication barriers. This is notable given that in 2020, one in eight aged‐care recipients had a first language other than English (Australian Institute of Health and Welfare, 2023b). Effective communication is shaped not only by the personal factors and characteristics of aged‐care recipients but also by those of their communication partners, including direct care staff. In Australia, 36% of personal care workers in residential care settings identify as being from culturally and linguistically diverse backgrounds (Australian Institute of Health and Welfare, 2023b), a factor that has been reported as a barrier to effective staff–recipient communication (McKenzie et al., 2024). Further, only 9% of identified articles related to intergenerational communication training. However, in Australia, over 50% of the direct care workforce in residential settings are aged under 40 years (Australian Department of Health and Aged Care, 2021). Similarly, 55% of Australian personal care workers in community or home‐based settings are aged under 50 years (Australian Department of Health and Aged Care, 2021). These statistics indicate that training addressing intergenerational communication differences is warranted. There is therefore a need for comprehensive CPT programs for aged‐care settings, that consider communication support needs arising from combinations of personal and environmental factors, as well as health or medical causes.

### Format of CPT programs

The most common mode of delivery for CPT was in‐person, group‐based training, which was delivered in a one‐off instance. This aligns with McKenzie et al. (2024) survey findings, where 48% of residential aged‐care workers indicated a preference for face‐to‐face training, particularly if the focus is communication. However, the feasibility of providing in‐person CPT in an Australian context must be considered due to the instability of the workforce, the availability of qualified and appropriate trainers, as well as geographical barriers. In Australia, there are over 400 000 direct care staff employed across all aged‐care settings (Australian Institute of Health and Welfare, 2023a). Between 2023 and 2024, attrition of aged‐care staff was approximately 25%, with one in four workers leaving the sector that year (Australian Institute of Health and Welfare, 2024b). High staff turnover is a complication for training in the sector more broadly (Australian Institute of Health and Welfare, 2023a; Cortie et al., 2024). Consequently, if CPT—and training generally—were delivered only in‐person, frequent training repetition would be required, increasing costs and resource demands for service providers. Given these challenges, consideration of alternative delivery models is warranted. There is a growing body of research exploring the effectiveness of both online and asynchronous CPT delivery modalities. For example, a recent study by Power et al. (2020) identified that the delivery of a short, online CPT intervention was effective in improving both student knowledge and attitudes towards communication disability. This suggests that online training modalities may be as effective as traditional in‐person methods (Power et al., 2020). A comprehensive CPT program that is online and/or asynchronous may be more feasible and effective within the identified constraints of the Australian aged‐care context.

A key issue identified in this review is that almost all interventionists were either clinicians or researchers (*n* = 70, 78%). Notably, speech pathologists were the most frequently reported professionals to deliver training. Given that speech pathologists are recognised experts in communication disability and communication partner training, they should play a significant role in the education and training of aged‐care workers (Speech Pathology Australia, 2015). However, there is a current shortage of speech pathologists throughout Australia in a range of settings (Jobs and Skills Australia, 2023), as well as a workforce shortage of skilled allied health professionals in regional, rural and remote locations (Cortie et al., 2024). In addition, aged‐care funding has been identified as inadequate for existing clinician interventions for people using aged‐care services (Allied Health Professions Australia, 2021, 2023; Meulenbroeks et al., 2023), and does not sufficiently fund allied health providers to deliver coaching, up‐skilling or in‐services to aged‐care staff (Allied Health Professions Australia, 2023; Speech Pathology Australia, 2011). It is unlikely to be feasible for speech pathologists to routinely implement and deliver CPT. Alternative implementation models must therefore be considered. For example, there is an emerging evidence‐base for train‐the‐trainer (Degen et al., 2022; Franzmann et al., 2016) and collaborative coaching (Douglas et al., 2023) models. As noted previously, online and asynchronous training models may also be an effective use of clinical resources for CPT training across the sector (Power et al., 2023; Williams et al., 2021). These delivery models may be more practical in the Australian context, given current workforce shortages and funding constraints for allied health expertise and training more broadly in the aged‐care sector.

Delivering in‐person training to aged‐care workers across a country as vast as Australia is challenging, given the many regional, rural and remote locations of aged‐care service providers (Hodgkin et al., 2017; Savy et al., 2017). In 2023, nearly 40% of all residential aged‐care services in Australia were located outside of metropolitan areas, with 21% in rural, remote or very remote areas (Australian Institute of Health and Welfare, 2023b). If CPT were to be provided via in‐person modalities, workers from non‐metropolitan locations may be disadvantaged by having to travel large distances to access essential training. In addition, in Australia, there are no nationally mandated minimum training requirements (Royal Commission into Aged Care Quality and Safety, 2021), so continuing professional development for staff, including associated costs, often falls to individual service providers (Martyn et al., 2022). However, rural and remote service providers face unique resource constraints, including increased operational costs (Baldwin et al., 2013), compared to their metropolitan counterparts. Therefore, it is crucial to consider the feasibility of various training modalities, to address the potential disparities in training opportunities available to care staff from different geographical locations (Hodgkin et al., 2017; Martyn et al., 2022; McKenzie et al., 2024; Savy et al., 2017). This also underscores that resource‐intensive training across services and locations remains a critical consideration for CPT delivery.

### Outcome measures

Our review demonstrates that the outcomes of CPT are measured in numerous ways. This aligns with Tessier et al. (2020) scoping review, where a lack of consistency in outcome measures used across disorder‐specific CPT programs was highlighted. Further, outcome measures do not adequately reflect the inherently dyadic nature of communication. Rotherham et al. (2024) found that existing CPT outcome measures rarely capture both trainee and care recipient outcomes. Most outcome measures identified in our review focused on changes in the trainee's knowledge, skills or attitudes, whereas partner‐ or patient‐specific measures were less frequently evaluated. To understand if CPT is effective in enhancing communication with and for aged‐care recipients, outcomes of CPT programs should measure not only the trainee's acquired competencies but also outcomes for the care recipient (Rotherham et al., 2024). Our review suggests further work is needed to develop consistent outcome measures for CPT programs. This could include establishing consensus on core outcomes for CPT programs designed for aged‐care settings. Core outcome sets have previously been developed in related areas (Mitchell et al., 2024; Volkmer et al., 2024; Wallace et al., 2023).

### Limitations

A key limitation of this review stems from limited access to the manuals for included CPT programs. Few articles included training manuals and materials as supplementary files or provided links to publicly available resources. Access to these materials would have enabled greater analysis of intervention components. Future CPT studies should consider the accessibility of training resources to allow accurate review, study replication and uptake and implementation of training in aged‐care settings.

This study used a scoping review methodology. Therefore, quality and effectiveness of identified CPT programs was not assessed. Future research using a systematic review methodology would provide a useful next step in terms of understand the impacts of CPT interventions for older adults with communication support needs.

Finally, given the diversity of terminology used to describe communication support needs and aged‐care services internationally, it is possible that some relevant papers may have been overlooked in this review. Furthermore, our search was limited to English‐language publications. Relevant research in alternative languages may have subsequently been excluded.

## CONCLUSION

Using a scoping review methodology, we identified and described CPT programs for direct support staff who work with older adults. Of the 90 articles identified, the majority focused on disorder‐specific CPT programs, such as dementia or aphasia communication training. There were no CPT programs that addressed intercultural communication support needs, and few addressed intergenerational communication. As aged‐care recipients have multifaceted, complex and intersecting communication support needs, existing programs may fall short of addressing the training needs of Australian aged‐care workers. Our analysis indicates a need for broad‐based CPT for staff that is disorder agnostic, aged‐care specific and includes the full range of possible communication support needs. CPT should also consider scalable delivery modes which are effective across the sector, and feasible given Australia's geographical spread and availability of qualified trainers. It should include outcome measures that incorporate the perspectives and impact on communication support partners: the older Australians using aged‐care services who have communication difficulties and support needs. In order to address the health, personal and environmental barriers to communication encountered in aged‐care environments, a fit‐for‐purpose CPT program developed in collaboration with key stakeholders is needed to ensure training addressing unmet needs is both feasible and acceptable for the intended end‐users.

## CONFLICT OF INTEREST STATEMENT

The authors have declared no conflicts of interest.

## Supporting information



Supporting Information

## Data Availability

The authors have provided supplementary materials containing data that support the findings of this scoping review. For further information, please contact the corresponding author.
